# Pixel Super-Resolution Phase Retrieval for Lensless On-Chip Microscopy via Accelerated Wirtinger Flow

**DOI:** 10.3390/cells11131999

**Published:** 2022-06-22

**Authors:** Yunhui Gao, Feng Yang, Liangcai Cao

**Affiliations:** State Key Laboratory of Precision Measurement Technology and Instruments, Department of Precision Instruments, Tsinghua University, Beijing 100084, China; gyh21@mails.tsinghua.edu.cn (Y.G.); yangfeng2020@mail.tsinghua.edu.cn (F.Y.)

**Keywords:** phase retrieval, pixel super-resolution, computational imaging, digital holography, Wirtinger flow, lensless imaging, whole slide imaging

## Abstract

Empowered by pixel super-resolution (PSR) and phase retrieval techniques, lensless on-chip microscopy opens up new possibilities for high-throughput biomedical imaging. However, the current PSR phase retrieval approaches are time consuming in terms of both the measurement and reconstruction procedures. In this work, we present a novel computational framework for PSR phase retrieval to address these concerns. Specifically, a sparsity-promoting regularizer is introduced to enhance the well posedness of the nonconvex problem under limited measurements, and Nesterov’s momentum is used to accelerate the iterations. The resulting algorithm, termed accelerated Wirtinger flow (AWF), achieves at least an order of magnitude faster rate of convergence and allows a twofold reduction in the measurement number while maintaining competitive reconstruction quality. Furthermore, we provide general guidance for step size selection based on theoretical analyses, facilitating simple implementation without the need for complicated parameter tuning. The proposed AWF algorithm is compatible with most of the existing lensless on-chip microscopes and could help achieve label-free rapid whole slide imaging of dynamic biological activities at subpixel resolution.

## 1. Introduction

The ever-increasing demand for information throughput toward biomedical and other engineering applications has strongly promoted recent developments in imaging techniques with a high space-bandwidth product [1,2,3]. Among these techniques, lensless on-chip microscopy has become an emerging solution by leveraging recent advances in sensor technology and computational power to overcome the inherent tradeoff between spatial resolution and field of view of the conventional point to-point imaging modality [4,5,6]. It captures a field-of-view as large as the sensor area with a resolution of a few microns in a single exposure, which makes it a competitive solution to rapid whole slide imaging for histopathology [7]. Compared with the lens-based counterparts, lensless on-chip microscopy also enables a compact and low-cost configuration [8,9]. Furthermore, its quantitative phase imaging capability allows for the label-free characterization of transparent or volumetric samples [10], which are commonly encountered in biomedical applications, such as pathology [11], inflammation [12], immunology [13], neuroscience [14], and cancer cell biology [15].

Despite their distinct advantages, lensless on-chip microscopy poses new challenges that need to be addressed. The phase information of the wavefield cannot be recorded due to the intensity-only response of the imaging sensors, and the high-frequency details beyond the Nyquist sampling limit are also lost during the measurement. Techniques to address these problems, namely phase retrieval [16] and pixel super-resolution (PSR) [17], play a pivotal role in high-fidelity and high-resolution holographic imaging. Phase retrieval aims to encode the phase of the wavefield by transferring it into intensity variations by physical means [18,19,20,21] and then numerically recover the phase (and amplitude) distributions via optimization algorithms [22,23,24]. Pixel super-resolution, on the other hand, aims to surpass the Nyquist sampling limit by similar physical encoding and numerical recovery procedures, pushing the resolution toward the diffraction limit [25,26,27,28,29,30].

In recent years, it has been recognized from a physical perspective that the missing phase and the undersampled high-frequency information can be both encoded into the intensity observations via diversity measurements [31,32,33], which can be implemented by varying the defocus distances [34,35,36], illumination wavelengths [37,38,39,40], modulation patterns [41,42,43], and probe positions [44,45,46], etc., making it possible for numerical recovery. On the algorithmic side, PSR phase retrieval can be achieved by a simple modification to the classical phase retrieval algorithms [47,48,49]. More recently, PSR phase retrieval has been recast as a standard optimization problem, which allows the use of off-the-shelf optimization tools, such as alternating projection and gradient descent algorithms [33].

Nevertheless, the range of applications of current PSR phase retrieval methods is primarily limited by the considerable time consumption during both the measurement and reconstruction stages. Due to the high dimension of the parameter space, it typically requires a larger number of diversity measurements to ensure the well posedness of the inverse problem and takes a longer time for iterative reconstruction compared with classical phase retrieval methods, yet acquisition and reconstruction speed is of vital importance in many imaging applications [50,51]. Additionally, the processing of the data is further complicated by manual parameter tuning due to the heuristic nature of the algorithms.

In this work, we introduce accelerated Wirtinger flow (AWF) as a unified framework for pixel super-resolution phase retrieval. Based on the proximal gradient method, AWF allows incorporating off-the-shelf regularization techniques to help improve imaging quality and reduce measurement number. Nesterov’s acceleration method is applied in the iterative reconstruction process, achieving at least an order of magnitude faster rate of convergence. Furthermore, the proposed algorithm features a prespecified step size, facilitating simple implementation to various system configurations.

## 2. Problem Formulation

### 2.1. Forward Model

**Figure 1:**
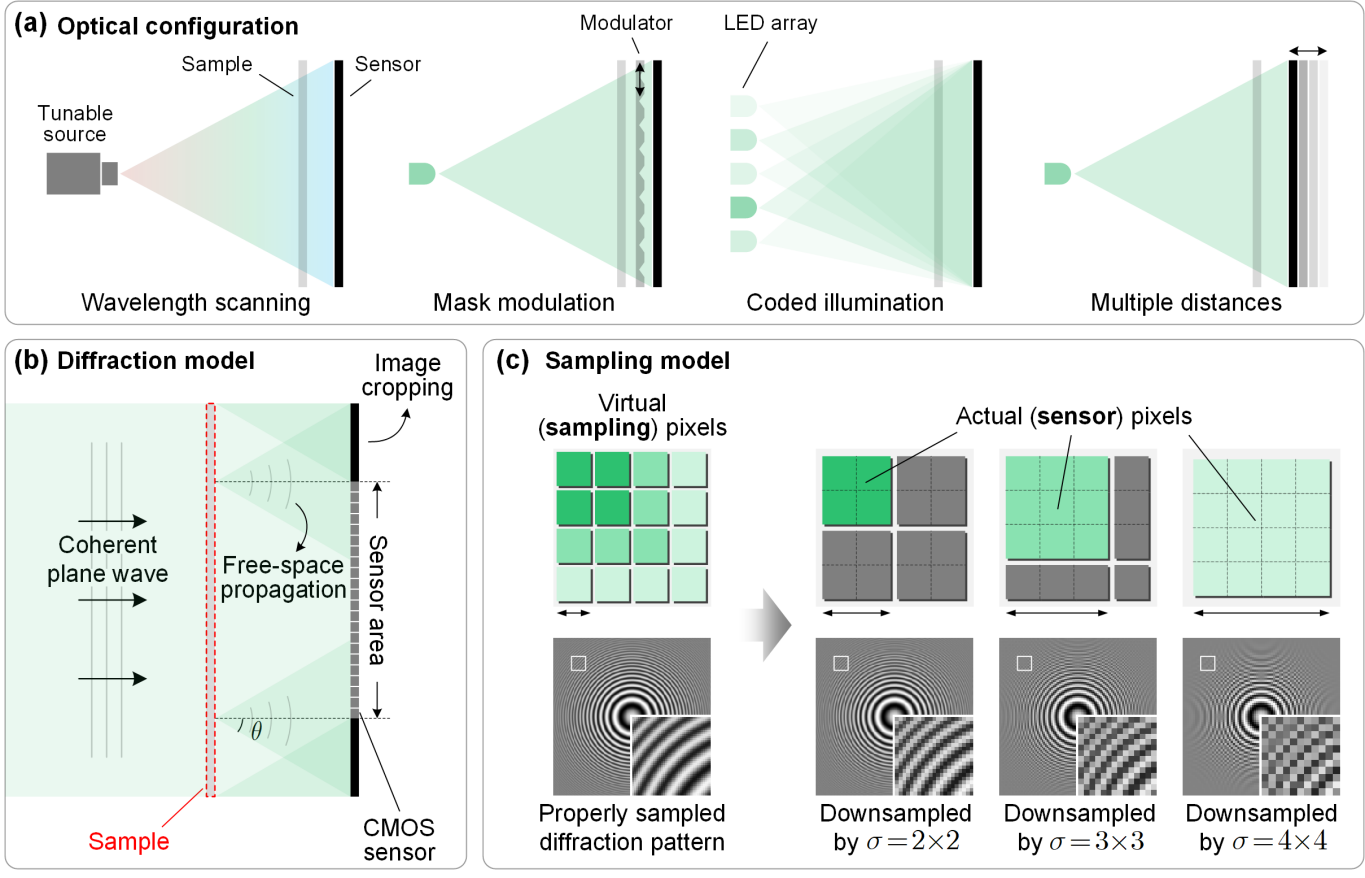
Forward model of a lensless on-chip microscope. (**a**) Typical optical configurations that can transfer the phase and subpixel information into the intensity variations at the sensor plane. (**b**) Diffraction model of the imaging system. Diffraction is calculated via the angular spectrum method, where the diffraction angle θ and the corresponding Fresnel kernel size are determined by the sampling frequency. (**c**) Sampling model of the sensor pixels.

**Figure 2:**
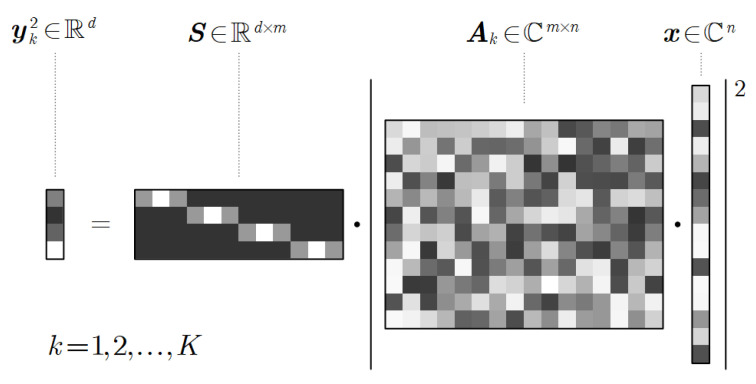
Intuitive illustration of the mathematical model.

Figure 1a shows some typical optical setups for a lensless on-chip microscope. The sample is illuminated by a coherent source. Diversity measurement is achieved by varying the physical parameters. The intensity of the coded wavefield is then recorded by the pixelated sensor. Therefore, the general forward model for lensless on-chip microscopes can be expressed as a linear transformation and a down-sampled quadratic measurement:

(1) $$y_{k}^{2}=S{\left|A_{k}x\right|}^{2},\;k=1,2,\cdots ,K,$$

where $x\in C^{n}$ represents the complex transmittance of the sample, $A_{k}\in C^{m\times n}$ denotes the sampling matrix for the *k*-th of out *K* diversity measurements, and $y_{k}^{2}\in R^{d}$ denotes the corresponding intensity image. $S\in R^{d\times m}$ with m=σd represents the down-sampling (pixel binning) operation of the sensor pixels, where σ is a positive integer referred to as the down-sampling ratio. The down-sampling operator performs a weighted sum of the subpixel intensities. Conceptual illustrations of the physical model and the mathematical model are shown in Figure 1 and Figure 2, respectively. Note that ${\left(\cdot \right)}^{2}$ and |·| are element-wise operators.

While the forward measurement matrix $A_{k}$ given in Equation (1) seems rather abstract, it in fact encapsulates a wide variety of physical processes. For example, when using defocus diversity or wavelength diversity measurements, $A_{k}$ may represent the free-space propagation with multiple distances or wavelengths. In the case of ptychography or modulation diversity, it may also incorporate coded illumination or mask modulation.

The introduction of the down-sampling matrix S makes the forward model of Equation (1) different from that of classical phase retrieval problems. It arises from the fact that all the photons incident upon a same pixel are converted into a single intensity signal. Mathematically, the intensity signal can be regarded as a weighted sum of the signals of the corresponding subpixels. In many works, uniform weights are adopted. Nevertheless, in practice, the fill factor of the sensor pixels is usually less than one, thus the weights for different subpixels may be different. A more accurate sampling model can be obtained by experimentally calibrating the intensity response of the sensor pixels, as was well done in [7]. Note that this down-sampling process is quite similar to the single pixel imaging model, despite the fact that we are using array sensors [52]. The model described by Equation (1) subsumes a special case the classical phase retrieval problem when σ=1 and S is an identity matrix.

### 2.2. Regularized Inversion

Based on the forward model of Equation (1), PSR phase retrieval can be formulated as a regularized inverse problem as follows:

(2) x^=argminx11112K∑k=1KSAkx2−yk22⏟F(x)+111λDx1⏟R(x),

where F(x) and R(x) are the data-fidelity function and the regularization function, respectively. ${∥\cdot ∥}_{p}$ denotes the $\ell_{p}$ vector norm. The data-fidelity function ensures the estimate x is consistent with the forward model. Considering the inherent ill posedness of PSR phase retrieval, an additional regularization term R(x) is introduced that encourages certain solutions based on prior knowledge of the sample. We use the anisotropic complex total variation (TV) in this work as an example, where $D\in R^{2n\times n}$ denotes the finite difference operator and λ>0 is a regularization parameter. When λ=0, Equation (2) reduces to the non-regularized case.

It should be noted that there exists various mathematically equivalent choices for the data-fidelity function. The same issue has been well studied in the case of classical phase retrieval. The *intensity-based* formulation aims to minimize the intensity residuals, which was adopted by many theoretical studies [24,53,54]. Another choice is the *amplitude-based* formulation [55,56,57,58]. It has been observed by many prior works that minimizing the lower-order amplitude-based fidelity function leads to faster convergence compared with minimizing the intensity-based one [59]. In analogy to the classical phase retrieval, the lower-order fidelity function in Equation (2) was adopted in [33] for PSR phase retrieval, whose superior performance was also experimentally verified.

The regularization term R(x) encourages certain solutions based on prior knowledge of the sample. In classical phase retrieval, chances of recovering the unknown sample rely heavily on the well posedness of the problem. Broadly speaking, there are two ways to ensure well posedness, that is, increasing the number of measurements, or incorporating signal priors [60]. In the case of PSR phase retrieval, it is generally more time consuming to acquire sufficient diversity data to suppress ambiguous solutions. As a result, to tackle the ill-posedness, we introduce another regularization term R(x) for the reconstruction problem. Apart from the total variation function adopted in this paper, the regularization function can take many other forms, such as BM3D [42,61,62,63] and deep denoiser priors [64,65].

## 3. Derivation of Algorithms

### 3.1. Accelerated Wirtinger Flow

The proximal gradient method is adopted for solving the non-smooth composite optimization problem of Equation (2), which proceeds by minimizing the two terms in an alternative manner [66]. Specifically, we apply a gradient update step with respect to the fidelity term, whose Wirtinger gradient is given by [33]

(3) $$\nabla_{x}F\left(x\right)=\frac{1}{2K}\sum_{k=1}^{K}A_{k}^{H}\mathrm{diag}\left(A_{k}x\right)S^{T}\left(1-\frac{y_{k}}{\sqrt{S{\left|A_{k}x\right|}^{2}}}\right),$$

where ${\left(\cdot \right)}^{T}$ and ${\left(\cdot \right)}^{H}$ denote the transpose and Hermitian operators, respectively. diag(·) puts the element of a vector onto the diagonal of a matrix. $\sqrt{\cdot }$ and ·/· are element-wise operators. Rigorously speaking, the fidelity function is not differentiable at points where $S|A_{k}{x|}^{2}$ has zero entries for some *k*. However, its non-smoothness can be addressed by assigning a certain value to the gradients at these points. The regularization term is updated via its proximity operator:

(4) $${\mathrm{prox}}_{\gamma R}\left(v\right)=\underset{x}{\mathrm{argmin}}\frac{1}{2\gamma }{∥x-v∥}_{2}^{2}+R\left(x\right),$$

where γ>0 denotes the step size. For the complex TV function, in particular, an efficient algorithm for calculating the proximal update has been proposed in [67]. We found that when the regularization parameter λ is relatively small (as is often the case), a single iteration of the inner loop is sufficient for good performance.

The basic proximal gradient algorithm, however, can be very slow in terms of convergence. Fortunately, relating the existing PSR phase retrieval algorithms with theoretically tractable optimization frameworks allows us to explore advanced techniques that help improve the algorithmic performance. In this work, we introduce the well-known Nesterov’s acceleration method as an example. It was originally proposed for minimization of smooth convex functions [68], and was later extended to the proximal gradient method [69]. Inspired by Nesterov’s method, some recent works have applied similar acceleration schemes to classical phase retrieval [70,71,72,73]. Following this line of research, we introduce a similar acceleration scheme to PSR phase retrieval, leading to the following iterates:

(5) $$\begin{array}{cc} v^{\left(t\right)}= & \;u^{\left(t-1\right)}-\gamma \nabla_{u}F\left(u^{\left(t-1\right)}\right), \end{array}$$

(6) $$\begin{array}{cc} x^{\left(t\right)}= & \;{\mathrm{prox}}_{\gamma R}\left(v^{\left(t\right)}\right), \end{array}$$

(7) $$\begin{array}{cc} u^{\left(t\right)}= & \;x^{\left(t\right)}+\beta_{t}\left(x^{\left(t\right)}-x^{\left(t-1\right)}\right), \end{array}$$

where t=1,2,⋯,T, γ>0 is the step size, and $u^{\left(0\right)}=x^{\left(0\right)}$. The algorithm is termed as accelerated Wirtinger flow when $\beta_{t}=t/\left(t+3\right)$ is used for the the extrapolation step of Equation (7), as is suggested by Nesterov’s method.

### 3.2. Convergence Analysis

We next provide general guidance for the step size selection of the AWF algorithm. Considering the nonconvexity of the PSR phase retrieval problem, we present a weaker theoretical result establishing the convergence of the non-accelerated Wirtinger flow algorithm, which is summarized by the following theorem. Nevertheless, we empirically observe a stable convergence behavior of the AWF algorithm using the same step size. A detailed proof of the theorem can be found in the Supplementary Materials document.

**Convergence** **Theorem** **1.** *The Wirtinger flow iterates of Equations (5)–(7) with $\beta_{t}\equiv 0$ converge to a stationary point using a fixed step size* γ *that satisfies*

(8) $$\left.\gamma \leq 2K/\sum_{k=1}^{K}\rho \left(A_{k}^{H}\mathrm{diag}\left(s\right)A_{k}\right),\right.$$

*where* $s=S^{T}\cdot 1$ *, and* ρ(·) *denotes the spectral radius.*

## 4. Experimental Results

### 4.1. System Configuration

**Figure 3:**
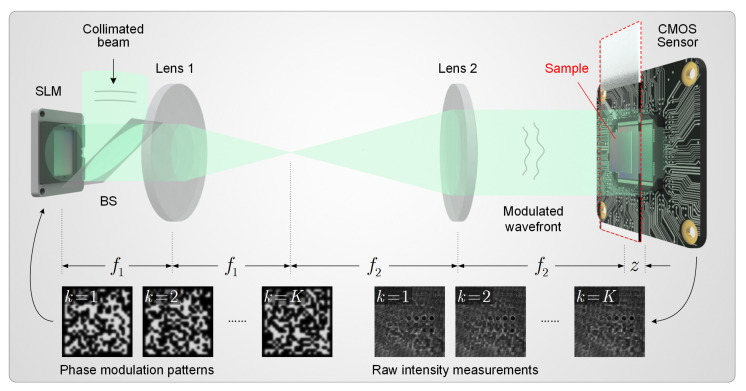
Lensless on-chip microscope based on phase modulation diversity, which we consider as an example in this work. BS is a beam splitter. $f_{1}$ and $f_{2}$ denote the focal lengths of Lens 1 and Lens 2, respectively. *z* denotes the sample-to-sensor distance.

To validate the proposed AWF method, we consider a particular holographic imaging model based on phase modulation diversity, as shown in Figure 3. A phase-only spatial light modulator (SLM) is placed at the conjugate plane of a 4f system with respect to the sample, generating phase diversity by varying the modulation patterns. The diffraction patterns of the modulated wavefield are recorded by a CMOS sensor, which is placed close to the sample. Using SLM for diversity measurement enables higher data acquisition speed compared with approaches that require mechanical displacements. Phase-only liquid crystal SLMs can typically achieve a frame rate of 60 Hz [74]. Thus, measurements can be completed in less than a few seconds.

Based on the optical setup, we now formulate the specific mathematical model and derive the step size for the algorithm. The sampling matrix $A_{k}$ can be divided into three linear operations, namely a phase-only modulation by the SLM $M_{k}\in C^{n\times n}\;\left(k=1,2,\cdots ,K\right)$, a free-space propagation $H\in C^{n\times n}$ which is implemented via circular convolution based on the angular spectrum method, and an image cropping operation due to the finite size of the sensor area $C\in R^{m\times n}$. That is, we have $A_{k}={\mathrm{CHM}}_{k}$. As for the down-sampling operator S, we assume a spatially uniform weight for different subpixel responses. A more accurate sampling model can be obtained by experimentally calibrating the intensity response of the sensor pixels. Based on the above modeling, one can easily verify that $\rho (A_{k}^{H}\mathrm{diag}\left(s\right)A_{k})\leq 1$ for all k=1,2,⋯,K (see Supplementary Materials document), which, according to the above theorem, implies a proper step size of γ=2. It is worth noticing that this specific choice of step size is nontrivial and generally applicable. For most optical settings, the measurement is passive in the sense that the sampling operators $A_{k}$ are non-expansive (i.e., $\rho (A_{k})\leq 1$) after proper normalization. Multiplication by a factor of two is due to the Wirtinger calculus [75].

To facilitate further applications and comparisons, a MATLAB implementation for the algorithms is available in [76]. All numerical experiments were conducted on a laptop computer equipped with an Intel Core i5 CPU at 1.60 GHz and 16 GB of memory. It takes approximately 20 s per iteration for reconstruction of a pixel super-resolved image of size n=1024×1024 using K=64 diversity images.

### 4.2. Simulation Studies

**Figure 4:**
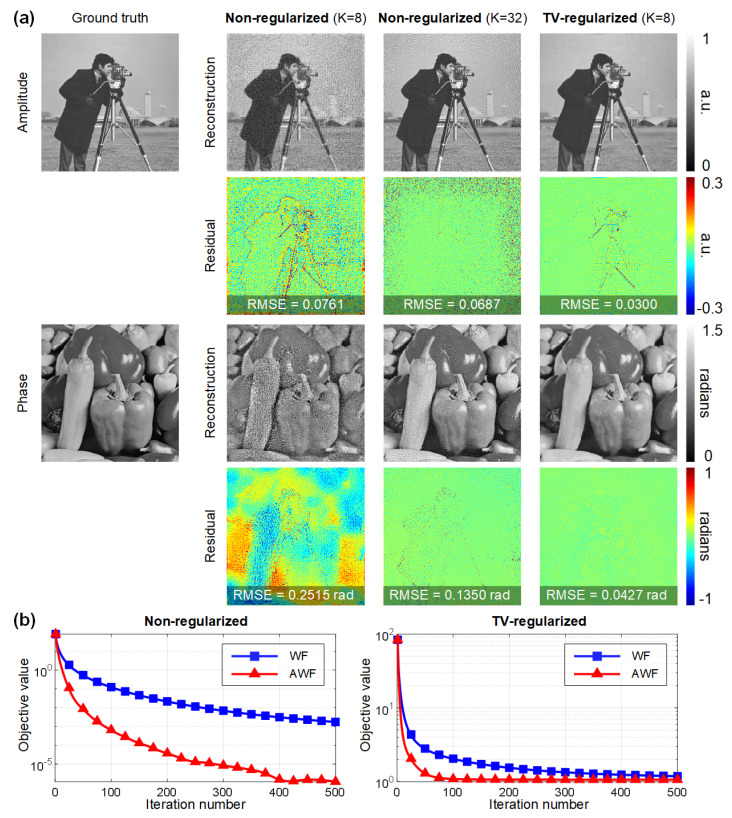
Simulation results. (**a**) Evaluation of the quality improvements by TV regularization. (**b**) Convergence curves of the AWF algorithm and the non-accelerated Wirtinger flow (WF) algorithm using K=8 diversity images.

Numerical studies were conducted to quantitatively study the performance improvements in terms of both reconstruction quality and convergence speed. The *Cameraman* image and the *Peppers* image were used to simulate the amplitude and phase distribution of a complex sample, respectively. Only K=8 intensity images with phase modulation diversity were used for PSR phase retrieval with an under-sampling ratio of σ=4×4=16, rendering the inverse problem severely ill-posed. Figure 4a shows the retrieved amplitude and phase via the AWF algorithm with and without the TV regularization term. The introduction of the regularizer helps significantly suppress the artifacts while preserving fine details of the image. Furthermore, the TV-regularized reconstruction with K=8 images also outperforms the non-regularized reconstruction with K=32 images, as is quantified by the root-mean-square errors (RMSEs). Figure 4b plots the convergence curves of the AWF algorithm and the non-accelerated proximal gradient algorithm. We observe that in both non-regularized and regularized cases, AWF exhibits at least an order of magnitude faster rate of convergence compared with the basic algorithm, which empirically demonstrates the effectiveness of Nesterov’s method for this nonconvex optimization problem.

### 4.3. Optical Experiments

Experimental data were collected from an inline holographic imaging system, where a 532 nm laser was used for coherent illumination, a phase-only reflective SLM (GAEA-2, HOLOEYE, Berlin, Germany) was used to generate phase patterns, and a CMOS sensor (QHY163M, pixel pitch 3.8 μm, QHYCCD, Beijing, China) was used to record the intensity images. Readers may refer to [41] for a detailed description of the system configuration. The phase response of the SLM was calibrated by a self-referenced interferometric method [77,78,79]. The phase modulation patterns were randomly generated and then Gaussian-filtered in order to introduce enough diversity while minimizing the crosstalk effect between adjacent SLM pixels.

**Figure 5:**
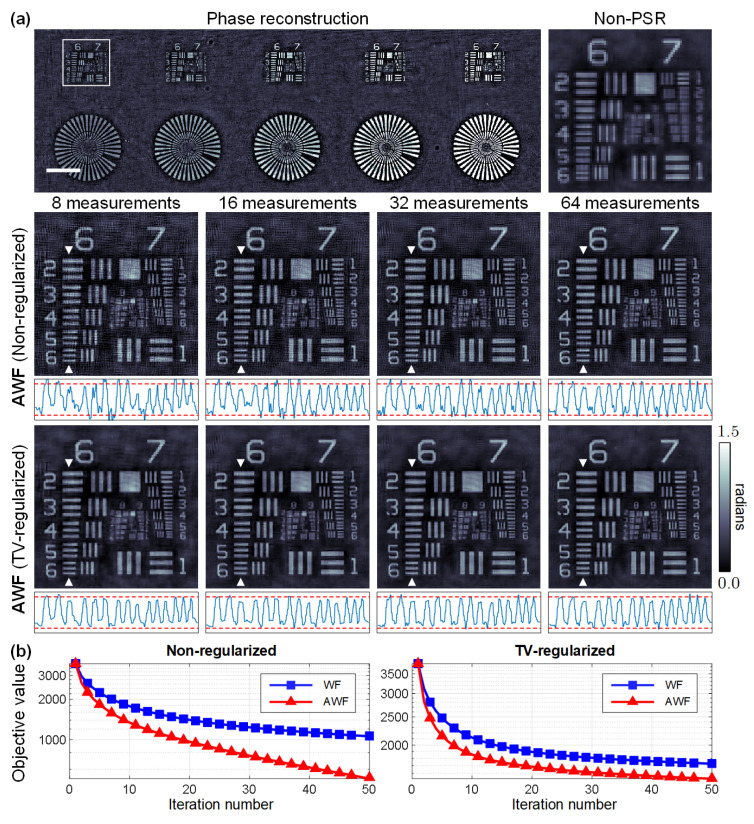
Experimental results. (**a**) Phase reconstruction of a quantitative phase target. The cross-sectional profiles are indicated by the triangular marks. The red doted lines indicate the ground truth phase induced by the structures. The scale bar is 200 μm. (**b**) Convergence curves of the algorithms using K=8 diversity images.

We first evaluated the proposed method via imaging a quantitative phase microscopy target (Benchmark Technologies, Lynnfield, MA, USA). The up-sampling ratio was set to σ=3×3, leading to a higher imaging resolution than the classical non-PSR phase retrieval method. Reconstruction via the non-regularized and the TV-regularized PSR phase retrieval models was implemented using K=8, 16, 32, and 64 intensity images, respectively. In the optical experiments, the artifacts arise not only from the measurement noise, but also from the modeling errors of the imaging system that are inevitable in practice. The TV regularization can help reduce the artifacts while preserving high-frequency details, as is visually demonstrated by the phase images and quantitatively verified by the cross-sectional phase profiles in Figure 5a. The ground truth values of the phase structures were calculated by φ=2πh(RI−1)/w, where *h* denotes the height of the structure, RI denotes the refractive index of the medium, and *w* denotes the illumination wavelength. Figure 5b indicates a similar improvement to the convergence rate using AWF compared with WF. Considering the inevitable modeling errors of the imaging system, the algorithms tend to converge earlier on experimental data.

**Figure 6:**
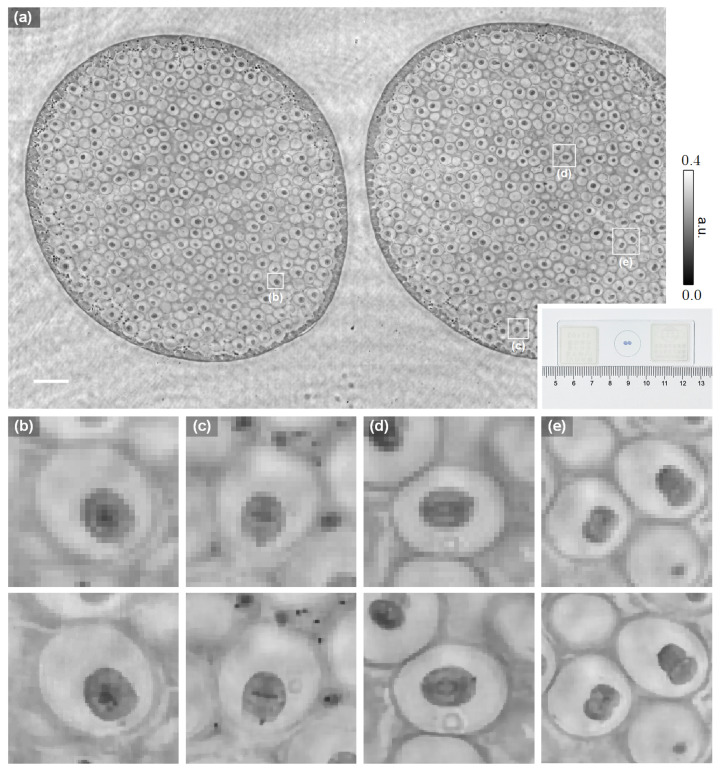
Experimental validation on biological samples.(**a**) Amplitude reconstruction of a section of the uterus of *parascaris equorum*. The inset shows an image of the stained tissue slide. (**b**–**e**) are the enlarged images of (**a**), corresponding to the prophase, metaphase, anaphase, and telophase of the mitosis, respectively. The upper and lower rows show the non-PSR and PSR reconstruction, respectively. The scale bar is 200 μm.

We further tested the resolving power of the proposed method on the imaging of a biological sample under the same optical settings. Figure 6 shows the imaging results of an iron-hematoxylin stained slide of the uterus of *parascaris equorum*. The piece-wise smoothness property of the TV regularizer helps suppress the noise and artifacts significantly, although the reconstruction quality may be further improved by more advanced image priors. The shape of the chromosomes can be resolved by the PSR phase retrieval method with high fidelity, from which one could easily recognize different phases of the mitosis. In prephase (Figure 6b), the chromatin condenses into chromosomes. In metaphase (Figure 6c), the chromosomes line up along the equatorial plane. In anaphase (Figure 6d), two sets of daughter chromosomes are pulled toward opposite ends of the cell. Finally, in telophase (Figure 6e), a new envelope forms around each set of separated daughter chromosomes, and cell division occurs. These biological activities, however, are not clearly revealed by the conventional non-PSR phase retrieval method because the distribution of the chromosomes cannot be directly resolved by the relatively large sensor pixels.

## 5. Conclusions

To conclude, we proposed AWF as a general pixel super-resolution phase retrieval framework for lensless on-chip microscopy that helps reduce time consumption during both the measurement and reconstruction procedures. To speed up the data acquisition, we introduced the TV regularizer to tackle the ill posedness of PSR phase retrieval. As is demonstrated by both simulated and experimental data, the introduction of the TV regularizer allows at least a twofold reduction in the number of intensity images while maintaining competitive resolution and quality. To speed up the iterative reconstruction, we applied Nesterov’s method to the nonconvex PSR phase retrieval problem. The accelerated algorithm converges at least an order of magnitude faster than the conventional one. On the theoretical side, we demonstrated through theoretical analyses that the lower-order fidelity function has favorable geometrical properties that ensure convergence of the Wirtinger flow iterates using a prespecified step size. Our findings extend previous results on classical phase retrieval to the PSR case, which may help bridge the gap between empirical and theoretical studies. The proposed algorithmic framework is generally applicable to the existing lensless on-chip microscopy platforms, and may thus facilitate a wide range of biomedical applications, such as whole slide histopathology and cell biology.

## Data Availability

The MATLAB code for AWF is available in [76]. Experimental data underlying the results presented in this paper may be obtained from the authors upon reasonable request.

## Supplementary Materials

The following supporting information can be downloaded at: https://www.mdpi.com/article/10.3390/cells11131999/s1. References [33,75,80,81] are cited in the Supplementary Materials.

## References

[1] Huang Z., Cao L. (2022). High bandwidth-utilization digital holographic multiplexing: An approach using Kramers–Kronig relations. Adv. Photonics Res..

[2] Pan A., Zuo C., Yao B. (2020). High-resolution and large field-of-view Fourier ptychographic microscopy and its applications in biomedicine. Rep. Prog. Phys..

[3] Park J., Brady D.J., Zheng G., Tian L., Gao L. (2021). Review of bio-optical imaging systems with a high space-bandwidth product. Adv. Photonics.

[4] Greenbaum A., Luo W., Khademhosseinieh B., Su T.W., Coskun A.F., Ozcan A. (2013). Increased space-bandwidth product in pixel super-resolved lensfree on-chip microscopy. Sci. Rep..

[5] Fan Y., Li J., Lu L., Sun J., Hu Y., Zhang J., Li Z., Shen Q., Wang B., Zhang R. (2021). Smart computational light microscopes (SCLMs) of smart computational imaging laboratory (SCILab). PhotoniX.

[6] Ozcan A., McLeod E. (2016). Lensless imaging and sensing. Annu. Rev. Biomed. Eng..

[7] Jiang S., Guo C., Song P., Zhou N., Bian Z., Zhu J., Wang R., Dong P., Zhang Z., Liao J. (2021). Resolution-enhanced parallel coded ptychography for high-throughput optical imaging. ACS Photonics.

[8] Sobieranski A.C., Inci F., Tekin H.C., Yuksekkaya M., Comunello E., Cobra D., Von Wangenheim A., Demirci U. (2015). Portable lensless wide-field microscopy imaging platform based on digital inline holography and multi-frame pixel super-resolution. Light Sci. Appl..

[9] Guo C., Liu X., Zhang F., Du Y., Zheng S., Wang Z., Zhang X., Kan X., Liu Z., Wang W. (2022). Lensfree on-chip microscopy based on single-plane phase retrieval. Opt. Express.

[10] Park Y., Depeursinge C., Popescu G. (2018). Quantitative phase imaging in biomedicine. Nat. Photonics.

[11] Lee K., Kim K., Jung J., Heo J., Cho S., Lee S., Chang G., Jo Y., Park H., Park Y. (2013). Quantitative phase imaging techniques for the study of cell pathophysiology: From principles to applications. Sensors.

[12] Bettenworth D., Bokemeyer A., Poremba C., Ding N.S., Ketelhut S., Lenz P., Kemper B. (2018). Quantitative phase microscopy for evaluation of intestinal inflammation and wound healing utilizing label-free biophysical markers. Histol. Histopathol..

[13] Yoon J., Jo Y., Kim M.h., Kim K., Lee S., Kang S.J., Park Y. (2017). Identification of non-activated lymphocytes using three-dimensional refractive index tomography and machine learning. Sci. Rep..

[14] Marquet P., Depeursinge C., Magistretti P.J. (2014). Review of quantitative phase-digital holographic microscopy: Promising novel imaging technique to resolve neuronal network activity and identify cellular biomarkers of psychiatric disorders. Neurophotonics.

[15] Kemper B., Carl D.D., Schnekenburger J., Bredebusch I., Schäfer M., Domschke W., von Bally G. (2006). Investigation of living pancreas tumor cells by digital holographic microscopy. J. Biomed. Opt..

[16] Shechtman Y., Eldar Y.C., Cohen O., Chapman H.N., Miao J., Segev M. (2015). Phase retrieval with application to optical imaging: A contemporary overview. IEEE Signal Process. Mag..

[17] Mudanyali O., McLeod E., Luo W., Greenbaum A., Coskun A.F., Hennequin Y., Allier C.P., Ozcan A. (2013). Wide-field optical detection of nanoparticles using on-chip microscopy and self-assembled nanolenses. Nat. Photonics.

[18] Zhang Y., Pedrini G., Osten W., Tiziani H.J. (2003). Whole optical wave field reconstruction from double or multi in-line holograms by phase retrieval algorithm. Opt. Express.

[19] Faulkner H.M.L., Rodenburg J.M. (2004). Movable aperture lensless transmission microscopy: A novel phase retrieval algorithm. Phys. Rev. Lett..

[20] Zhang F., Pedrini G., Osten W. (2007). Phase retrieval of arbitrary complex-valued fields through aperture-plane modulation. Phys. Rev. A.

[21] Bao P., Zhang F., Pedrini G., Osten W. (2008). Phase retrieval using multiple illumination wavelengths. Opt. Lett..

[22] Gerchberg R.W., Saxton W. (1971). A practical algorithm for the determination of phase from image and diffraction plane pictures. Optik.

[23] Fienup J.R. (1982). Phase retrieval algorithms: A comparison. Appl. Opt..

[24] Candès E.J., Li X., Soltanolkotabi M. (2015). Phase retrieval via Wirtinger flow: Theory and algorithms. IEEE Trans. Inf. Theory.

[25] Greenbaum A., Feizi A., Akbari N., Ozcan A. (2013). Wide-field computational color imaging using pixel super-resolved on-chip microscopy. Opt. Express.

[26] Bishara W., Su T.W., Coskun A.F., Ozcan A. (2010). Lensfree on-chip microscopy over a wide field-of-view using pixel super-resolution. Opt. Express.

[27] Greenbaum A., Ozcan A. (2012). Maskless imaging of dense samples using pixel super-resolution based multi-height lensfree on-chip microscopy. Opt. Express.

[28] Luo W., Greenbaum A., Zhang Y., Ozcan A. (2015). Synthetic aperture-based on-chip microscopy. Light Sci. Appl..

[29] Fournier C., Jolivet F., Denis L., Verrier N., Thiebaut E., Allier C., Fournel T. (2017). Pixel super-resolution in digital holography by regularized reconstruction. Appl. Opt..

[30] Lee H., Kim J., Kim J., Jeon P., Lee S.A., Kim D. (2021). Noniterative sub-pixel shifting super-resolution lensless digital holography. Opt. Express.

[31] Luo W., Zhang Y., Göröcs Z., Feizi A., Ozcan A. (2016). Propagation phasor approach for holographic image reconstruction. Sci. Rep..

[32] Zhang J., Sun J., Chen Q., Zuo C. (2020). Resolution analysis in a lens-free on-chip digital holographic microscope. IEEE Trans. Comput. Imaging.

[33] Gao Y., Cao L. (2021). Generalized optimization framework for pixel super-resolution imaging in digital holography. Opt. Express.

[34] Shen C., Guo C., Geng Y., Tan J., Liu S., Liu Z. (2018). Noise-robust pixel-super-resolved multi-image phase retrieval with coherent illumination. J. Opt..

[35] Guo C., Zhang F., Zhang X., Kan X., Tan J., Liu S., Liu Z. (2020). Lensfree super-resolved imaging based on adaptive Wiener filter and guided phase retrieval algorithm. J. Opt..

[36] Jiang S., Guo C., Hu P., Hu D., Song P., Wang T., Bian Z., Zhang Z., Zheng G. (2021). High-throughput lensless whole slide imaging via continuous height-varying modulation of a tilted sensor. Opt. Lett..

[37] Luo W., Zhang Y., Feizi A., Göröcs Z., Ozcan A. (2016). Pixel super-resolution using wavelength scanning. Light Sci. Appl..

[38] Song P., Wang R., Zhu J., Wang T., Bian Z., Zhang Z., Hoshino K., Murphy M., Jiang S., Guo C. (2020). Super-resolved multispectral lensless microscopy via angle-tilted, wavelength-multiplexed ptychographic modulation. Opt. Lett..

[39] Wu X., Sun J., Zhang J., Lu L., Chen R., Chen Q., Zuo C. (2021). Wavelength-scanning lensfree on-chip microscopy for wide-field pixel-super-resolved quantitative phase imaging. Opt. Lett..

[40] Wang Q., Ma J., Su P. (2022). A multi-wavelength phase retrieval with multi-strategy for lensfree onchip holography. Front. Photonics.

[41] Gao Y., Cao L. (2020). High-fidelity pixel-super-resolved complex field reconstruction via adaptive smoothing. Opt. Lett..

[42] Katkovnik V., Shevkunov I., Petrov N.V., Egiazarian K. (2017). Computational super-resolution phase retrieval from multiple phase-coded diffraction patterns: Simulation study and experiments. Optica.

[43] Guo Y., Guo R., Qi P., Zhou Y., Zhang Z., Zheng G., Zhong J. (2022). Robust multi-angle structured illumination lensless microscopy via illumination angle calibration. Opt. Lett..

[44] Zhang H., Bian Z., Jiang S., Liu J., Song P., Zheng G. (2019). Field-portable quantitative lensless microscopy based on translated speckle illumination and sub-sampled ptychographic phase retrieval. Opt. Lett..

[45] Jiang S., Zhu J., Song P., Guo C., Bian Z., Wang R., Huang Y., Wang S., Zhang H., Zheng G. (2020). Wide-field, high-resolution lensless on-chip microscopy via near-field blind ptychographic modulation. Lab Chip.

[46] Lv W., Zhang J., Chen H., Yang D., Ruan T., Zhu Y., Tao Y., Shi Y. (2022). Resolution-enhanced ptychography framework with an equivalent upsampling and precise position. Appl. Opt..

[47] Zhang J., Sun J., Chen Q., Li J., Zuo C. (2017). Adaptive pixel-super-resolved lensfree in-line digital holography for wide-field on-chip microscopy. Sci. Rep..

[48] Rivenson Y., Wu Y., Wang H., Zhang Y., Feizi A., Ozcan A. (2016). Sparsity-based multi-height phase recovery in holographic microscopy. Sci. Rep..

[49] Zhang F., Guo C., Zhai Y., Tan J., Liu S., Tan C., Chen H., Liu Z. (2020). A noise-robust multi-intensity phase retrieval method based on structural patch decomposition. J. Opt..

[50] Shimobaba T., Sato Y., Miura J., Takenouchi M., Ito T. (2008). Real-time digital holographic microscopy using the graphic processing unit. Opt. Express.

[51] Tian L., Li X., Ramchandran K., Waller L. (2014). Multiplexed coded illumination for Fourier ptychography with an LED array microscope. Biomed. Opt. Express.

[52] Edgar M.P., Gibson G.M., Padgett M.J. (2019). Principles and prospects for single-pixel imaging. Nat. Photonics.

[53] Zhang H., Chi Y., Liang Y. Provable non-convex phase retrieval with outliers: Median truncated Wirtinger flow. Proceedings of the 33rd International Conference on Machine Learning, PMLR 48.

[54] Isernia T., Leone G., Pierri R. (1996). Radiation pattern evaluation from near-field intensities on planes. IEEE Trans. Antennas Propag..

[55] Zhang H., Liang Y. (2016). Reshaped Wirtinger flow for solving quadratic systems of equations. Adv. Neural Inf. Process. Syst..

[56] Wang G., Giannakis G.B., Eldar Y.C. (2018). Solving systems of random quadratic equations via truncated amplitude flow. IEEE Trans. Inf. Theory.

[57] Wang G., Giannakis G.B., Saad Y., Chen J. (2018). Phase retrieval via reweighted amplitude flow. IEEE Trans. Signal Process..

[58] Wang G., Giannakis G.B. Solving random systems of quadratic equations via truncated generalized gradient flow. Proceedings of the 30th International Conference on Neural Information Processing Systems.

[59] Yeh L.H., Dong J., Zhong J., Tian L., Chen M., Tang G., Soltanolkotabi M., Waller L. (2015). Experimental robustness of Fourier ptychography phase retrieval algorithms. Opt. Express.

[60] Grohs P., Koppensteiner S., Rathmair M. (2020). Phase retrieval: Uniqueness and stability. SIAM Rev..

[61] Kocsis P., Shevkunov I., Katkovnik V., Egiazarian K. (2020). Single exposure lensless subpixel phase imaging: Optical system design, modelling, and experimental study. Opt. Express.

[62] Shevkunov I., Katkovnik V., Petrov N.V., Egiazarian K. (2018). Super-resolution microscopy for biological specimens: Lensless phase retrieval in noisy conditions. Biomed. Opt. Express.

[63] Kocsis P., Shevkunov I., Katkovnik V., Rekola H., Egiazarian K. (2021). Single-shot pixel super-resolution phase imaging by wavefront separation approach. Opt. Express.

[64] Chang X., Bian L., Gao Y., Cao L., Suo J., Zhang J. (2022). Plug-and-play pixel super-resolution phase retrieval for digital holography. Opt. Lett..

[65] Chang X., Jiang S., Zheng G., Bian L. (2022). Deep distributed optimization for blind diffuser-modulation ptychography. Opt. Lett..

[66] Parikh N., Boyd S. (2014). Proximal algorithms. Found. Trends Optim..

[67] Gao Y., Cao L. (2021). A Complex Constrained Total Variation Image Denoising Algorithm with Application to Phase Retrieval. arXiv.

[68] Nesterov Y.E. (1983). A method for solving the convex programming problem with convergence rate O (1/k 2). Dokl. Akad. Nauk SSSR.

[69] Beck A., Teboulle M. (2009). A fast iterative shrinkage-thresholding algorithm for linear inverse problems. SIAM J. Imaging Sci..

[70] Xu R., Soltanolkotabi M., Haldar J.P., Unglaub W., Zusman J., Levi A.F., Leahy R.M. (2018). Accelerated Wirtinger flow: A fast algorithm for ptychography. arXiv.

[71] Bostan E., Soltanolkotabi M., Ren D., Waller L. Accelerated Wirtinger flow for multiplexed Fourier ptychographic microscopy. Proceedings of the 2018 25th IEEE International Conference on Image Processing (ICIP).

[72] Zhou Y., Zhang H., Liang Y. Geometrical properties and accelerated gradient solvers of non-convex phase retrieval. Proceedings of the 2016 54th Annual Allerton Conference on Communication, Control, and Computing (Allerton).

[73] Fabian Z., Haldar J., Leahy R., Soltanolkotabi M. 3D phase retrieval at nano-scale via accelerated Wirtinger flow. Proceedings of the 2020 28th European Signal Processing Conference (EUSIPCO).

[74] https://holoeye.com/gaea-4k-phase-only-spatial-light-modulator/.

[75] Kreutz-Delgado K. (2009). The complex gradient operator and the CR-Calculus. arXiv.

[76] https://github.com/THUHoloLab/pixel-super-resolution-phase-retrieval.

[77] Li R., Cao L. (2019). Progress in phase calibration for liquid crystal spatial light modulators. Appl. Sci..

[78] Gao Y., Li R., Cao L. (2019). Self-referenced multiple-beam interferometric method for robust phase calibration of spatial light modulator. Opt. Express.

[79] Li R., Gao Y., Cao L. (2020). In situ calibration for a phase-only spatial light modulator based on digital holography. Opt. Eng..

[80] Horn R.A., Johnson C.R. (2012). Matrix Analysis.

[81] Beck A. (2017). First-Order Methods in Optimization.

