# Supplementary material for: Pixel Super-Resolution Phase Retrieval for Lensless On-Chip Microscopy via Accelerated Wirtinger Flow
Source: Cells. 2022 Jun 22;11(13):1999. doi: 10.3390/cells11131999 (PMC9265759; doi:10.3390/cells11131999)
Supplement: Supplementary file 1 [file cells-11-01999-s001.zip › cells-1759172-supplementary.pdf]

Supplementary Materials: Pixel super-resolution phase retrieval for lensless on-chip microscopy via accelerated Wirtinger flow

Yunhui Gao [1], Feng Yang [1] and Liangcai Cao [1,*]

1. Step size selection

We briefly discuss the step size selection based on the convergence theorem for our particular optical configuration, as described in the main text. The imaging model can be divided into three linear operations, namely a phase-only modulation by the spatial light modulator $\boldsymbol{M}_k \in \mathbb{C}^{n\times n}$ $(k = 1, 2, \ldots, K)$, a free-space propagation $\boldsymbol{H} \in \mathbb{C}^{n\times n}$ which is implemented via circular convolution based on the angular spectrum model, and an image cropping operation due to the finite size of the sensor area $\boldsymbol{C} \in \mathbb{R}^{m\times n}$. Therefore, the sampling matrix can be expressed as

$$\boldsymbol{A}_k = \boldsymbol{C}\boldsymbol{H}\boldsymbol{M}_k. \tag{1}$$

Note that we have $\boldsymbol{M}_k^{\mathsf{H}}\boldsymbol{M}_k = \boldsymbol{I}$ for all $k = 1, 2, \ldots, K$ and $\boldsymbol{H}^{\mathsf{H}}\boldsymbol{H} = \boldsymbol{I}$, where $\boldsymbol{I}$ denotes the identity matrix. We further assume a uniform intensity response for all subpixels, that is, we set all the weights to one. Thus, $\mathrm{diag}(\boldsymbol{s}) = \mathrm{diag}(\boldsymbol{S}^{\mathsf{T}} \cdot \boldsymbol{1}) = \boldsymbol{I}$. One can easily verify that the spectral radius of $\boldsymbol{A}_k^{\mathsf{H}}\mathrm{diag}(\boldsymbol{s})\boldsymbol{A}_k$ is upper-bounded as follows:

$$\begin{aligned}\rho(\boldsymbol{A}_k^{\mathsf{H}}\mathrm{diag}(\boldsymbol{s})\boldsymbol{A}_k) = \rho(\boldsymbol{A}_k^{\mathsf{H}}\boldsymbol{A}_k) = \|\boldsymbol{A}_k\|_2^2 = \left(\max_{\boldsymbol{x}\neq\boldsymbol{0}} \frac{\|\boldsymbol{A}_k\boldsymbol{x}\|_2}{\|\boldsymbol{x}\|_2}\right)^2 = \left(\max_{\boldsymbol{x}\neq\boldsymbol{0}} \frac{\|\boldsymbol{C}\boldsymbol{H}\boldsymbol{M}_k\boldsymbol{x}\|_2}{\|\boldsymbol{x}\|_2}\right)^2 \\ \overset{\boldsymbol{u}=\boldsymbol{H}\boldsymbol{M}_k\boldsymbol{x}}{\underset{(a)}{=\!=}} \left(\max_{\boldsymbol{u}\neq\boldsymbol{0}} \frac{\|\boldsymbol{C}\boldsymbol{u}\|_2}{\|\boldsymbol{u}\|_2}\right)^2 \leq 1,\end{aligned} \tag{2}$$

where (a) holds because $\boldsymbol{H}\boldsymbol{M}_k$ is unitary. Therefore,

$$2K \Bigg/ \sum_{k=1}^{K} \rho\left(\boldsymbol{A}_k^{\mathsf{H}}\mathrm{diag}(\boldsymbol{s})\boldsymbol{A}_k\right) \leq 2K/K = 2, \tag{3}$$

and according to the convergence theorem, $\gamma = 2$ is proper step size.

2. Proof of Convergence

2.1. Preliminaries

In this Section, we present some intermediate results regarding matrix analysis, which would be helpful for proving the convergence theorem below.

Lemma 1 (Properties of (semi-)definite matrices [1]). *Given matrices* $\boldsymbol{P} \in \mathbb{C}^{n\times n}$, $\boldsymbol{Q} \in \mathbb{C}^{n\times n}$, *and* $\boldsymbol{R} \in \mathbb{C}^{n\times n}$. *The following holds:*

(a) $\boldsymbol{P} \succ \boldsymbol{Q} \Rightarrow \boldsymbol{R}^{\mathsf{H}}\boldsymbol{P}\boldsymbol{R} \succ \boldsymbol{R}^{\mathsf{H}}\boldsymbol{Q}\boldsymbol{R}$,

(b) $\boldsymbol{P} \succeq \boldsymbol{Q} \succ \boldsymbol{0} \Rightarrow \boldsymbol{Q}^{-1} \succeq \boldsymbol{P}^{-1} \succ \boldsymbol{0}$.

Lemma 2 (Schur Complement [1]). *Given a* $2n \times 2n$ *Hermitian matrix:*

$$\boldsymbol{P} = \begin{pmatrix} \boldsymbol{P}_{11} & \boldsymbol{P}_{12} \\ \boldsymbol{P}_{21} & \boldsymbol{P}_{22} \end{pmatrix}, \tag{4}$$

where each block is of size $n \times n$, *and we have* $\boldsymbol{P}_{11}^{\mathsf{H}} = \boldsymbol{P}_{11}$, $\boldsymbol{P}_{22}^{\mathsf{H}} = \boldsymbol{P}_{22}$, *and* $\boldsymbol{P}_{12}^{\mathsf{H}} = \boldsymbol{P}_{21}$. *Then*

$$\boldsymbol{P} \succ \boldsymbol{0} \Leftrightarrow \boldsymbol{P}_{11} \succ \boldsymbol{0} \quad \text{and} \quad \boldsymbol{P}_{22} - \boldsymbol{P}_{21}\boldsymbol{P}_{11}^{-1}\boldsymbol{P}_{12} \succ \boldsymbol{0}. \tag{5}$$

Lemma 3. *Suppose* $\boldsymbol{P} = (p_{ij}) \in \mathbb{R}^{n\times n}$ *is a positive symmetric matrix. That is,* $\boldsymbol{P}^{\mathsf{T}} = \boldsymbol{P}$*, and* $p_{ij} \leq 0$ *for all* $1 \leq i \leq n$, $1 \leq j \leq n$*. Then for any vector* $\boldsymbol{v} \in \mathbb{C}^n$*,*

$$\mathrm{diag}(\boldsymbol{v})\boldsymbol{P}\mathrm{diag}(\bar{\boldsymbol{v}}) \succeq \mathrm{diag}\Big(\boldsymbol{P}|\boldsymbol{v}|^2\Big), \tag{6}$$

where $\overline{(\cdot)}$ *denotes the complex conjugate.*

Proof. Given any $\boldsymbol{u} = (u_i) \in \mathbb{C}^n$, we have

$$\begin{aligned}
&\boldsymbol{u}^{\mathsf{H}}\Big(\mathrm{diag}\Big(\boldsymbol{P}|\boldsymbol{v}|^2\Big) - \mathrm{diag}(\boldsymbol{v})\boldsymbol{P}\mathrm{diag}(\bar{\boldsymbol{v}})\Big)\boldsymbol{u} \\
&= \boldsymbol{u}^{\mathsf{H}}\mathrm{diag}\Big(\boldsymbol{P}|\boldsymbol{v}|^2\Big)\boldsymbol{u} - \boldsymbol{u}^{\mathsf{H}}\mathrm{diag}(\boldsymbol{v})\boldsymbol{P}\mathrm{diag}(\bar{\boldsymbol{v}})\boldsymbol{u} \\
&= \sum_{i=1}^{n}\left(\sum_{j=1}^{n} p_{ij}|v_j|^2\right)|u_i|^2 - \sum_{i=1}^{n}\sum_{j=1}^{n} p_{ij}v_i\bar{v}_j u_i\bar{u}_j \\
&= \sum_{i=1}^{n}\sum_{j>i}^{n} p_{ij}\Big(|v_j|^2|u_i|^2 - p_{ij}v_i\bar{v}_j u_i\bar{u}_j - p_{ij}v_j\bar{v}_i u_j\bar{u}_i + |v_i|^2|u_j|^2\Big) \\
&= \sum_{i=1}^{n}\sum_{j>i}^{n} p_{ij}|\bar{v}_j u_i - v_i\bar{u}_j|^2 \\
&\geq 0.
\end{aligned} \tag{7}$$

Therefore, by definition, $\mathrm{diag}(\boldsymbol{P}|\boldsymbol{v}|^2) - \mathrm{diag}(\boldsymbol{v})\boldsymbol{P}\mathrm{diag}(\bar{\boldsymbol{v}})$ is positive-semidefinite, which completes the proof. □

Lemma 4. *Given a matrix* $\boldsymbol{P} \in \mathbb{C}^{n\times n}$*, and a scalar* $\varepsilon > 0$*,*

$$\boldsymbol{P}\Big(\varepsilon\boldsymbol{I} + \boldsymbol{P}^{\mathsf{H}}\boldsymbol{P}\Big)^{-1}\boldsymbol{P}^{\mathsf{H}} \prec \boldsymbol{I}. \tag{8}$$

Proof. Suppose the singular value decomposition of $\boldsymbol{P}$ is given by $\boldsymbol{P} = \boldsymbol{U}\boldsymbol{\Sigma}\boldsymbol{V}^{\mathsf{H}}$, where $\boldsymbol{U} \in \mathbb{C}^{n\times n}$ and $\boldsymbol{V} \in \mathbb{C}^{n\times n}$ are unitary matrices, and $\boldsymbol{\Sigma} = \mathrm{diag}(\boldsymbol{z})$ is a real-valued diagonal matrix. Then, we have

$$\varepsilon\boldsymbol{I} + \boldsymbol{P}^{\mathsf{H}}\boldsymbol{P} = \varepsilon\boldsymbol{I} + \boldsymbol{V}\boldsymbol{\Sigma}^2\boldsymbol{V}^{\mathsf{H}} = \boldsymbol{V}\mathrm{diag}\Big(\varepsilon\mathbf{1} + \boldsymbol{z}^2\Big)\boldsymbol{V}^{\mathsf{H}}. \tag{9}$$

That is, $\boldsymbol{P}^{\mathsf{H}}\boldsymbol{P}$ is diagonalizable with real-valued non-negative eigenvalues $z_1^2, z_2^2, \cdots, z_n^2$. $\varepsilon\boldsymbol{I} + \boldsymbol{P}^{\mathsf{H}}\boldsymbol{P}$ is nonsingular and its inverse is given by

$$\Big(\varepsilon\boldsymbol{I} + \boldsymbol{P}^{\mathsf{H}}\boldsymbol{P}\Big)^{-1} = \boldsymbol{V}\mathrm{diag}\left(\frac{\mathbf{1}}{\varepsilon\mathbf{1} + \boldsymbol{z}^2}\right)\boldsymbol{V}^{\mathsf{H}}. \tag{10}$$

Thus, we arrive at the result:

$$\boldsymbol{P}\Big(\varepsilon\boldsymbol{I} + \boldsymbol{P}^{\mathsf{H}}\boldsymbol{P}\Big)^{-1}\boldsymbol{P}^{\mathsf{H}} = \boldsymbol{U}\mathrm{diag}\left(\frac{\boldsymbol{z}^2}{\varepsilon\mathbf{1} + \boldsymbol{z}^2}\right)\boldsymbol{U}^{\mathsf{H}} \prec \boldsymbol{U}\boldsymbol{U}^{\mathsf{H}} = \boldsymbol{I}, \tag{11}$$

which completes the proof. □

2.2. Gradient and Hessian Calculation

In this Section, we derive the complex gradient and Hessian of the data-fidelity function based on the CR-calculus [2]. The CR-calculus extends the complex derivative to the general non-analytic functions, providing a powerful tool to analyze real-valued

functions over complex-valued variables. We consider the fidelity term with respect to the k-th intensity image:

$$F_k(\boldsymbol{x}) = \frac{1}{2}\left\| \sqrt{S|\boldsymbol{A}_k\boldsymbol{x}|^2} - \boldsymbol{y}_k \right\|_2^2. \quad (12)$$

The CR-calculus regards the complex variable $\boldsymbol{x}$ and its conjugate $\bar{\boldsymbol{x}}$ as independent variables. Thus, the fidelity function F_k should be interpreted as a function over the pair of conjugate vectors $\hat{\boldsymbol{x}} = [\boldsymbol{x}^{\mathsf{T}}, \bar{\boldsymbol{x}}^{\mathsf{T}}]^{\mathsf{T}}$, and the gradient is $\nabla F_k = [\nabla_{\boldsymbol{x}} F_k^{\mathsf{T}}, \nabla_{\bar{\boldsymbol{x}}} F_k^{\mathsf{T}}]^{\mathsf{T}}$. Nevertheless, to keep notations consistent, we still denote the function as $F_k(\boldsymbol{x})$. The same applies to other functions as well. The first-order partial derivatives of F_k with respect to $\boldsymbol{x}$ and $\bar{\boldsymbol{x}}$ have been derived in [3], which are

$$\frac{\partial F_k(\boldsymbol{x})}{\partial \boldsymbol{x}} = \frac{1}{2}\left(\boldsymbol{1} - \frac{\boldsymbol{y}_k}{\sqrt{S|\boldsymbol{A}_k\boldsymbol{x}|^2}}\right)^{\mathsf{T}} S\mathrm{diag}\left(\overline{\boldsymbol{A}_k\boldsymbol{x}}\right)\boldsymbol{A}_k, \quad (13)$$

$$\frac{\partial F_k(\boldsymbol{x})}{\partial \bar{\boldsymbol{x}}} = \frac{1}{2}\left(\boldsymbol{1} - \frac{\boldsymbol{y}_k}{\sqrt{S|\boldsymbol{A}_k\boldsymbol{x}|^2}}\right)^{\mathsf{T}} S\mathrm{diag}(\boldsymbol{A}_k\boldsymbol{x})\bar{\boldsymbol{A}}_k. \quad (14)$$

It should be noted that, the above Wirtinger derivatives are not well-defined for $\boldsymbol{x} \in Z_k$, where

$$Z_k \stackrel{\text{def}}{=} \left\{\boldsymbol{x} \in \mathbb{C}^n : \exists i, \text{ s.t. } \left(S|\boldsymbol{A}_k\boldsymbol{x}|^2\right)_i = 0\right\}. \quad (15)$$

Thus, we only consider $\boldsymbol{x} \in \mathbb{C}^n \backslash Z$, where $Z \stackrel{\text{def}}{=} Z_1 \cup Z_2 \cup \cdots \cup Z_K$. The complex Hessian is defined as

$$\nabla^2 F_k(\boldsymbol{x}) = \boldsymbol{H}_{\hat{\boldsymbol{x}}\hat{\boldsymbol{x}}} = \begin{pmatrix} \boldsymbol{H}_{\boldsymbol{x}\boldsymbol{x}} & \boldsymbol{H}_{\bar{\boldsymbol{x}}\boldsymbol{x}} \\ \boldsymbol{H}_{\boldsymbol{x}\bar{\boldsymbol{x}}} & \boldsymbol{H}_{\bar{\boldsymbol{x}}\bar{\boldsymbol{x}}} \end{pmatrix}, \quad (16)$$

where the four matrices are given as follows:

$$\begin{aligned}
\boldsymbol{H}_{\boldsymbol{x}\boldsymbol{x}} =& \frac{\partial}{\partial \boldsymbol{x}}\left(\frac{\partial F_k(\boldsymbol{x})}{\partial \boldsymbol{x}}\right)^{\mathsf{H}} \\
=& \frac{1}{2}\frac{\partial}{\partial \boldsymbol{x}}\left(\boldsymbol{A}_k^{\mathsf{H}}\mathrm{diag}(\boldsymbol{A}_k\boldsymbol{x})S^{\mathsf{T}}\left(\boldsymbol{1} - \frac{\boldsymbol{y}_k}{\sqrt{S|\boldsymbol{A}_k\boldsymbol{x}|^2}}\right)\right) \\
=& \frac{1}{2}\frac{\partial}{\partial \boldsymbol{x}}\left(\boldsymbol{A}_k^{\mathsf{H}}\mathrm{diag}(\boldsymbol{s})\boldsymbol{A}_k\boldsymbol{x} - \boldsymbol{A}_k^{\mathsf{H}}\mathrm{diag}(\boldsymbol{A}_k\boldsymbol{x})S^{\mathsf{T}}\frac{\boldsymbol{y}_k}{\sqrt{S|\boldsymbol{A}_k\boldsymbol{x}|^2}}\right) \\
=& \frac{1}{2}\boldsymbol{A}_k^{\mathsf{H}}\mathrm{diag}(\boldsymbol{s})\boldsymbol{A}_k - \frac{1}{2}\boldsymbol{A}_k^{\mathsf{H}}\mathrm{diag}\left(S^{\mathsf{T}}\frac{\boldsymbol{y}_k}{\sqrt{S|\boldsymbol{A}_k\boldsymbol{x}|^2}}\right)\boldsymbol{A}_k \\
&+ \frac{1}{4}\boldsymbol{A}_k^{\mathsf{H}}\mathrm{diag}(\boldsymbol{A}_k\boldsymbol{x})S^{\mathsf{T}}\mathrm{diag}\left(\frac{\boldsymbol{y}_k}{\left(S|\boldsymbol{A}_k\boldsymbol{x}|^2\right)^{\frac{3}{2}}}\right)S\mathrm{diag}(\overline{\boldsymbol{A}_k\boldsymbol{x}})\boldsymbol{A}_k,
\end{aligned} \quad (17)$$

$$\begin{aligned}
\boldsymbol{H}_{\bar{\boldsymbol{x}}\boldsymbol{x}} =& \frac{\partial}{\partial \bar{\boldsymbol{x}}}\left(\frac{\partial F_k(\boldsymbol{x})}{\partial \boldsymbol{x}}\right)^{\mathsf{H}} \\
=& \frac{1}{2}\frac{\partial}{\partial \bar{\boldsymbol{x}}}\left(\boldsymbol{A}_k^{\mathsf{H}}\mathrm{diag}(\boldsymbol{s})\boldsymbol{A}_k\boldsymbol{x} - \boldsymbol{A}_k^{\mathsf{H}}\mathrm{diag}(\boldsymbol{A}_k\boldsymbol{x})S^{\mathsf{T}}\frac{\boldsymbol{y}_k}{\sqrt{S|\boldsymbol{A}_k\boldsymbol{x}|^2}}\right) \\
=& \boldsymbol{0} - \frac{1}{2}\boldsymbol{A}_k^{\mathsf{H}}\mathrm{diag}(\boldsymbol{A}_k\boldsymbol{x})S^{\mathsf{T}}\frac{\partial}{\partial \bar{\boldsymbol{x}}}\left(\frac{\boldsymbol{y}_k}{\sqrt{S|\boldsymbol{A}_k\boldsymbol{x}|^2}}\right)
\end{aligned}$$

$$=\frac{1}{4}\boldsymbol{A}_k^{\mathsf{H}}\text{diag}(\boldsymbol{A}_k\boldsymbol{x})\boldsymbol{S}^{\mathsf{T}}\text{diag}\left(\frac{\boldsymbol{y}_k}{\left(\boldsymbol{S}|\boldsymbol{A}_k\boldsymbol{x}|^2\right)^{\frac{3}{2}}}\right)\boldsymbol{S}\text{diag}(\boldsymbol{A}_k\boldsymbol{x})\bar{\boldsymbol{A}}_k, \tag{18}$$

$$\boldsymbol{H}_{\boldsymbol{x}\bar{\boldsymbol{x}}}=\frac{\partial}{\partial \boldsymbol{x}}\left(\frac{\partial F_k(\boldsymbol{x})}{\partial \bar{\boldsymbol{x}}}\right)^{\mathsf{H}}=\boldsymbol{H}_{\bar{\boldsymbol{x}}\boldsymbol{x}}^{\mathsf{H}}$$

$$=\frac{1}{4}\boldsymbol{A}_k^{\mathsf{T}}\text{diag}(\overline{\boldsymbol{A}_k\boldsymbol{x}})\boldsymbol{S}^{\mathsf{T}}\text{diag}\left(\frac{\boldsymbol{y}_k}{\left(\boldsymbol{S}|\boldsymbol{A}_k\boldsymbol{x}|^2\right)^{\frac{3}{2}}}\right)\boldsymbol{S}\text{diag}(\overline{\boldsymbol{A}_k\boldsymbol{x}})\boldsymbol{A}_k, \tag{19}$$

$$\boldsymbol{H}_{\bar{\boldsymbol{x}}\bar{\boldsymbol{x}}}=\frac{\partial}{\partial \bar{\boldsymbol{x}}}\left(\frac{\partial F_k(\boldsymbol{x})}{\partial \bar{\boldsymbol{x}}}\right)^{\mathsf{H}}=\boldsymbol{H}_{\boldsymbol{x}\boldsymbol{x}}^{\mathsf{H}}$$

$$=\frac{1}{2}\boldsymbol{A}_k^{\mathsf{T}}\text{diag}(\boldsymbol{s})\bar{\boldsymbol{A}}_k-\frac{1}{2}\boldsymbol{A}_k^{\mathsf{T}}\text{diag}\left(\boldsymbol{S}^{\mathsf{T}}\frac{\boldsymbol{y}_k}{\sqrt{\boldsymbol{S}|\boldsymbol{A}_k\boldsymbol{x}|^2}}\right)\bar{\boldsymbol{A}}_k$$

$$+\frac{1}{4}\boldsymbol{A}_k^{\mathsf{T}}\text{diag}(\overline{\boldsymbol{A}_k\boldsymbol{x}})\boldsymbol{S}^{\mathsf{T}}\text{diag}\left(\frac{\boldsymbol{y}_k}{\left(\boldsymbol{S}|\boldsymbol{A}_k\boldsymbol{x}|^2\right)^{\frac{3}{2}}}\right)\boldsymbol{S}\text{diag}(\boldsymbol{A}_k\boldsymbol{x})\bar{\boldsymbol{A}}_k. \tag{20}$$

In the above derivation, we let $\boldsymbol{s}=\boldsymbol{S}^{\mathsf{T}}\cdot\mathbf{1}$.

2.3. Lipschitz Bound for the Wirtinger Gradient

In this Section, we prove that the gradient of $F(\boldsymbol{x})$ is upper Lipschitz bounded by a constant. This is a particularly useful property of the amplitude-based fidelity term, enabling us to use prespecified algorithm parameters while ensuring convergence.

Lemma 5 (Lipschitz Bound). *For any $\boldsymbol{x}\in\mathbb{C}^n\backslash Z$, the Lipschitz constant L for the gradient of the data-fidelity function $\nabla F(\boldsymbol{x})$ is bounded above:*

$$L\leq\frac{1}{2K}\sum_{k=1}^{K}\rho\left(\boldsymbol{A}_k^{\mathsf{H}}\text{diag}(\boldsymbol{s})\boldsymbol{A}_k\right), \tag{21}$$

where $\rho(\cdot)$ denotes the spectral radius.

Proof. We only need to prove that for all $1\leq k\leq K$, and for any $\boldsymbol{x}\in\mathbb{C}^n\backslash Z$, the gradient of the k-th data-fidelity term ∇F_k is Lipschitz continuous with Lipschitz constant L_k bounded above:

$$L_k\leq\frac{1}{2}\rho\left(\boldsymbol{A}_k^{\mathsf{H}}\text{diag}(\boldsymbol{s})\boldsymbol{A}_k\right), \tag{22}$$

which is equivalent to proving that for any $\tau>(1/2)\rho\left(\boldsymbol{A}_k^{\mathsf{H}}\text{diag}(\boldsymbol{s})\boldsymbol{A}_k\right)$, we have

$$\boldsymbol{G}\equiv\tau\boldsymbol{I}-\boldsymbol{H}_{\boldsymbol{c}\boldsymbol{c}}=\begin{pmatrix}\tau\boldsymbol{I}-\boldsymbol{H}_{\boldsymbol{x}\boldsymbol{x}} & -\boldsymbol{H}_{\bar{\boldsymbol{x}}\boldsymbol{x}}\\ -\boldsymbol{H}_{\boldsymbol{x}\bar{\boldsymbol{x}}} & \tau\boldsymbol{I}-\boldsymbol{H}_{\bar{\boldsymbol{x}}\bar{\boldsymbol{x}}}\end{pmatrix}\succ\mathbf{0}. \tag{23}$$

Let $\varepsilon=\tau-(1/2)\rho\left(\boldsymbol{A}_k^{\mathsf{H}}\text{diag}(\boldsymbol{s})\boldsymbol{A}_k\right)>0$, we have

$$\boldsymbol{G}_{11}=\left(\tau\boldsymbol{I}-\frac{1}{2}\boldsymbol{A}_k^{\mathsf{H}}\text{diag}(\boldsymbol{s})\boldsymbol{A}_k\right)+\frac{1}{2}\boldsymbol{A}_k^{\mathsf{H}}\text{diag}\left(\boldsymbol{S}^{\mathsf{T}}\frac{\boldsymbol{y}_k}{\sqrt{\boldsymbol{S}|\boldsymbol{A}_k\boldsymbol{x}|^2}}\right)\boldsymbol{A}_k$$

$$-\frac{1}{4}\boldsymbol{A}_k^{\mathsf{H}}\text{diag}(\boldsymbol{A}_k\boldsymbol{x})\boldsymbol{S}^{\mathsf{T}}\text{diag}\left(\frac{\boldsymbol{y}_k}{(\boldsymbol{S}|\boldsymbol{A}_k\boldsymbol{x}|)^{\frac{3}{2}}}\right)\boldsymbol{S}\text{diag}(\overline{\boldsymbol{A}_k\boldsymbol{x}})\boldsymbol{A}_k$$

$$\succ \varepsilon \boldsymbol{I} + \frac{1}{4}\boldsymbol{A}_k^{\mathsf{H}}\mathrm{diag}(\boldsymbol{A}_k\boldsymbol{x})\boldsymbol{S}^{\mathsf{T}}\mathrm{diag}\left(\frac{\boldsymbol{y}_k}{\left(\boldsymbol{S}|\boldsymbol{A}_k\boldsymbol{x}|^2\right)^{\frac{3}{2}}}\right)\boldsymbol{S}\mathrm{diag}(\overline{\boldsymbol{A}_k\boldsymbol{x}})\boldsymbol{A}_k, \tag{24}$$

where the inequality holds because

$$\tau \boldsymbol{I} - \frac{1}{2}\boldsymbol{A}_k^{\mathsf{H}}\mathrm{diag}(\boldsymbol{s})\boldsymbol{A}_k \succ \varepsilon \boldsymbol{I}, \tag{25}$$

and

$$\boldsymbol{A}_k^{\mathsf{H}}\mathrm{diag}\left(\boldsymbol{S}^{\mathsf{T}}\frac{\boldsymbol{y}_k}{\sqrt{\boldsymbol{S}|\boldsymbol{A}_k\boldsymbol{x}|^2}}\right) \succeq \boldsymbol{A}_k^{\mathsf{H}}\mathrm{diag}(\boldsymbol{A}_k\boldsymbol{x})\boldsymbol{S}^{\mathsf{T}}\mathrm{diag}\left(\frac{\boldsymbol{y}_k}{\left(\boldsymbol{S}|\boldsymbol{A}_k\boldsymbol{x}|^2\right)^{\frac{3}{2}}}\right)\boldsymbol{S}\mathrm{diag}(\overline{\boldsymbol{A}_k\boldsymbol{x}})\boldsymbol{A}_k. \tag{26}$$

Notice that the above equation follows directly from Lemma 3 by letting $\boldsymbol{v} = \boldsymbol{A}\boldsymbol{x}$ and $\boldsymbol{P} = \boldsymbol{S}^{\mathsf{T}}\mathrm{diag}\Big(\boldsymbol{y}_k/(\boldsymbol{S}|\boldsymbol{A}_k\boldsymbol{x}|^2)^{3/2}\Big)\boldsymbol{S}$ and using Lemma 1.a. Then, using Lemma 1.a again, we have

$$\boldsymbol{G}_{21}\boldsymbol{G}_{11}^{-1}\boldsymbol{G}_{12} \preceq \boldsymbol{G}_{21}\left(\varepsilon \boldsymbol{I} + \frac{1}{4}\boldsymbol{A}_k^{\mathsf{H}}\mathrm{diag}(\boldsymbol{A}_k\boldsymbol{x})\boldsymbol{S}^{\mathsf{T}}\mathrm{diag}\left(\frac{\boldsymbol{y}_k}{\left(\boldsymbol{S}|\boldsymbol{A}_k\boldsymbol{x}|^2\right)^{\frac{3}{2}}}\right)\boldsymbol{S}\mathrm{diag}(\overline{\boldsymbol{A}_k\boldsymbol{x}})\boldsymbol{A}_k\right)^{-1}\boldsymbol{G}_{12}. \tag{27}$$

Let $\boldsymbol{P} = (1/2)\mathrm{diag}\Big(\boldsymbol{y}_k/(\boldsymbol{S}|\boldsymbol{A}_k\boldsymbol{x}|^2)^{3/2}\Big)^{1/2}\boldsymbol{S}\mathrm{diag}(\overline{\boldsymbol{A}_k\boldsymbol{x}})\boldsymbol{A}_k$ and use Lemma 1.b, we have

$$\begin{aligned}
\boldsymbol{G}_{21}\boldsymbol{G}_{11}^{-1}\boldsymbol{G}_{12} &\preceq \frac{1}{4}\boldsymbol{A}_k^{\mathsf{T}}\mathrm{diag}(\overline{\boldsymbol{A}_k\boldsymbol{x}})\boldsymbol{S}^{\mathsf{T}}\mathrm{diag}\left(\frac{\boldsymbol{y}_k}{\left(\boldsymbol{S}|\boldsymbol{A}_k\boldsymbol{x}|^2\right)^{\frac{3}{2}}}\right)^{\frac{1}{2}} \\
&\quad\times \boldsymbol{P}\Big(\varepsilon \boldsymbol{I} + \boldsymbol{P}^{\mathsf{H}}\boldsymbol{P}\Big)^{-1}\boldsymbol{P}^{\mathsf{H}}\mathrm{diag}\left(\frac{\boldsymbol{y}_k}{\left(\boldsymbol{S}|\boldsymbol{A}_k\boldsymbol{x}|^2\right)^{\frac{3}{2}}}\right)^{\frac{1}{2}}\boldsymbol{S}\mathrm{diag}(\boldsymbol{A}_k\boldsymbol{x})\bar{\boldsymbol{A}}_k \\
&\prec \frac{1}{4}\boldsymbol{A}_k^{\mathsf{T}}\mathrm{diag}(\overline{\boldsymbol{A}_k\boldsymbol{x}})\boldsymbol{S}^{\mathsf{T}}\mathrm{diag}\left(\frac{\boldsymbol{y}_k}{\left(\boldsymbol{S}|\boldsymbol{A}_k\boldsymbol{x}|^2\right)^{\frac{3}{2}}}\right)\boldsymbol{S}\mathrm{diag}(\boldsymbol{A}_k\boldsymbol{x})\bar{\boldsymbol{A}}_k \\
&\prec \frac{1}{4}\boldsymbol{A}_k^{\mathsf{T}}\mathrm{diag}(\overline{\boldsymbol{A}_k\boldsymbol{x}})\boldsymbol{S}^{\mathsf{T}}\mathrm{diag}\left(\frac{\boldsymbol{y}_k}{\left(\boldsymbol{S}|\boldsymbol{A}_k\boldsymbol{x}|^2\right)^{\frac{3}{2}}}\right)\boldsymbol{S}\mathrm{diag}(\boldsymbol{A}_k\boldsymbol{x})\bar{\boldsymbol{A}}_k + \tau \boldsymbol{I} - \frac{1}{2}\boldsymbol{A}_k^{\mathsf{T}}\mathrm{diag}(\boldsymbol{s})\bar{\boldsymbol{A}}_k \\
&= \boldsymbol{G}_{22}.
\end{aligned} \tag{28}$$

Therefore, according to Lemma 2, $\boldsymbol{G}$ is positive-definite. This implies that for $\boldsymbol{x} \in \mathbb{C}^n \backslash Z_k$ the Lipschitz constant L_k of ∇F_k is upper-bounded by

$$L_k \leq \frac{1}{2}\rho\Big(\boldsymbol{A}_k^{\mathsf{H}}\mathrm{diag}(\boldsymbol{s})\boldsymbol{A}_k\Big). \tag{29}$$

Thus, for $\boldsymbol{x} \in \mathbb{C}^n \backslash Z$ the Lipschitz constant of ∇F satisfies

$$L = \frac{1}{K}\sum_{k=1}^{K} L_k \leq \frac{1}{2K}\sum_{k=1}^{K}\rho\Big(\boldsymbol{A}_k^{\mathsf{H}}\mathrm{diag}(\boldsymbol{s})\boldsymbol{A}_k\Big), \tag{30}$$

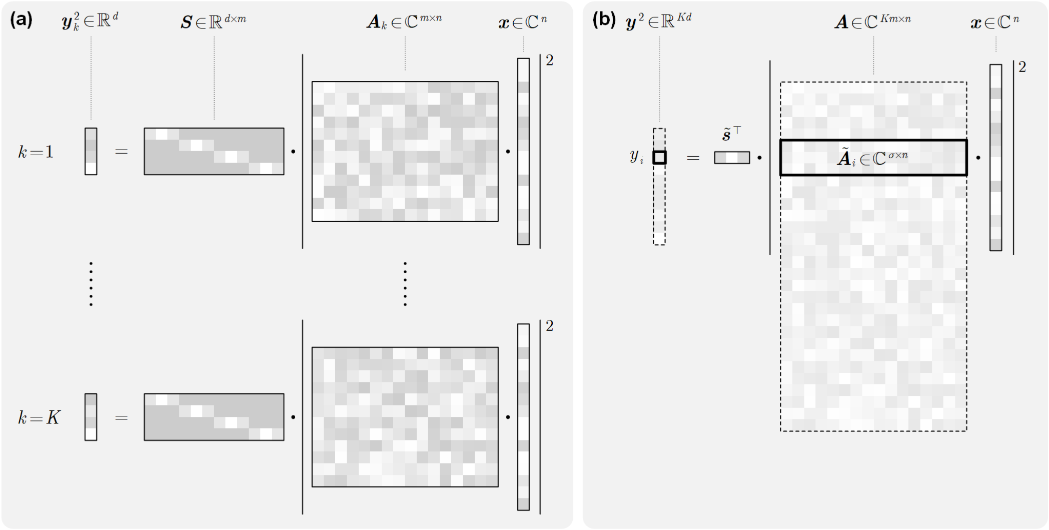

Figure S1. Illustration of two equivalent forward model formulations for PSR phase retrieval. (a) The image-wise formulation adopted in the main text. (b) The element-wise formulation adopted here for proof of convergence.

which completes the proof. □

2.4. Convergence of the Basic Algorithms

In this Section, we present the proof of convergence for the global gradient descent algorithm and the global proximal gradient algorithm with a constant step size. The main difficulty of the phase retrieval problem lies in the non-differentiability of the data-fidelity term at certain *nonsmooth points*, where the gradients are not well-defined.

For convenience of illustration, we adopt an equivalent formulation of the fidelity term, which is expressed in a element-wise form as

$$F(\boldsymbol{x}) = \frac{1}{2K}\sum_{i=1}^{M}\left(\sqrt{\tilde{\boldsymbol{s}}^{\top}|\tilde{\boldsymbol{A}}_i\boldsymbol{x}|^2} - y_i\right)^2 = \sum_{i=1}^{M} f_i(\boldsymbol{x}), \tag{31}$$

where $M = dK$, $\tilde{\boldsymbol{A}}_i \in \mathbb{C}^{\sigma\times n}\,(i = 1,2,\ldots,M)$ are the matrices extracted from $\boldsymbol{A}_k\,(k = 1,2,\ldots,K)$, and $\tilde{\boldsymbol{s}} \in \mathbb{R}^{\sigma}$ is the weight vector for the subpixels. f_i is defined as

$$f_i(\boldsymbol{x}) \overset{\text{def}}{=} \frac{1}{2K}\left(\sqrt{\tilde{\boldsymbol{s}}^{\top}|\tilde{\boldsymbol{A}}_i\boldsymbol{x}|^2} - y_i\right)^2. \tag{32}$$

A conceptual illustration for this formulation is shown in Fig. S1. Discontinuity of the gradient appears at points where for some $1 \le i \le M$, we have

$$\tilde{\boldsymbol{A}}_i\boldsymbol{x} = \boldsymbol{0}, \quad \tilde{\boldsymbol{A}}_i \neq \boldsymbol{0}, \quad \text{and} \quad y_i \neq 0. \tag{33}$$

We refer to any $\boldsymbol{x}$ that satisfies the above condition as a nonsmooth point.

Lemma 6. *Given any $\boldsymbol{z} \in \mathbb{C}^n$, the fidelity function $F(\boldsymbol{x})$ is upper-bounded by a quadratic function $Q(\boldsymbol{x})$:*

$$F(\boldsymbol{x}) \le Q(\boldsymbol{x}) \overset{\text{def}}{=} F(\boldsymbol{z}) + \nabla F(\boldsymbol{z})^{\mathsf{H}}(\hat{\boldsymbol{x}} - \hat{\boldsymbol{z}}) + \frac{L}{2}\|\hat{\boldsymbol{x}} - \hat{\boldsymbol{z}}\|_2^2. \tag{34}$$

Proof. Let $\Delta\boldsymbol{x} = \boldsymbol{x} - \boldsymbol{z}$, then either of the two following cases occurs:

1) The line between $\boldsymbol{x}$ and $\boldsymbol{z}$ does not pass through any nonsmooth points, i.e., $\boldsymbol{z} + \alpha\Delta\boldsymbol{x} \in \mathbb{C}^n\backslash Z$, $\forall\alpha \in [0,1]$, or $\boldsymbol{x}$ and $\boldsymbol{z}$ lie in the same nonsmooth subspace, i.e., $\boldsymbol{z} + \alpha\Delta\boldsymbol{x} \in$

Z, $\forall \alpha \in [0,1]$, the result is obtained directly according to the multivariate Taylor expansion of F:

$$\begin{aligned} F(\boldsymbol{x}) =& F(\boldsymbol{z}) + \nabla F(\boldsymbol{z})^{\mathsf{H}}(\hat{\boldsymbol{x}} - \hat{\boldsymbol{z}}) + \frac{1}{2}(\hat{\boldsymbol{x}} - \hat{\boldsymbol{z}})^{\mathsf{H}} \nabla^2 F(\boldsymbol{u})(\hat{\boldsymbol{x}} - \hat{\boldsymbol{z}}) \\ \leq& F(\boldsymbol{z}) + \nabla F(\boldsymbol{z})^{\mathsf{H}}(\hat{\boldsymbol{x}} - \hat{\boldsymbol{z}}) + \frac{L}{2}\|\hat{\boldsymbol{x}} - \hat{\boldsymbol{z}}\|_2^2 \\ =& Q(\boldsymbol{x}), \end{aligned} \tag{35}$$

where $\boldsymbol{u}$ is a convex combination of $\boldsymbol{x}$ and $\boldsymbol{z}$.

2) The line between $\boldsymbol{x}$ and $\boldsymbol{z}$ passes through a finite number of nonsmooth points. For simplicity, we consider the case of a single nonsmooth point indexed by j, that is, we have

$$\tilde{\boldsymbol{s}}^{\mathsf{T}} |\tilde{\boldsymbol{A}}_j(\boldsymbol{x} + \alpha^\star \Delta \boldsymbol{x})|^2 = 0, \tag{36}$$

for some $0 < \alpha^\star < 1$. The fidelity function can be written as a function over α for any point that lies on the line between $\boldsymbol{x}$ ad $\boldsymbol{z}$:

$$\begin{aligned} g(\alpha) =& F(\boldsymbol{x} + \alpha \Delta \boldsymbol{x}) \\ =& \sum_{i=1, i\neq j}^{M} f_i(\boldsymbol{x}) + f_j(\boldsymbol{x}) \\ =& \sum_{i=1, i\neq j}^{M} f_i(\boldsymbol{x}) + \frac{1}{2K}\left(\sqrt{\tilde{\boldsymbol{s}}^{\mathsf{T}} |\tilde{\boldsymbol{A}}_j(\boldsymbol{x} + \alpha \Delta \boldsymbol{x})|^2} - y_j\right)^2 \\ =& \sum_{i=1, i\neq j}^{M} f_i(\boldsymbol{x}) + \frac{1}{2K}\left(|\alpha - \alpha^\star|\sqrt{\tilde{\boldsymbol{s}}^{\mathsf{T}} |\tilde{\boldsymbol{A}}_j \Delta \boldsymbol{x}|^2} - y_j\right)^2. \end{aligned} \tag{37}$$

According to 1), for any $0 \leq \alpha \leq \alpha^\star$, g is upper-bounded by h:

$$h(\alpha) = Q(\boldsymbol{x} + \alpha \Delta \boldsymbol{x}). \tag{38}$$

We now prove that for any $\alpha^\star < \alpha < 1$, g is also upper-bounded by h, which is straightforward:

$$\begin{aligned} g(\alpha) =& \sum_{i=1, i\neq j}^{M} f_i(\boldsymbol{x}) + \frac{1}{2K}\left(|\alpha - \alpha^\star|\sqrt{\tilde{\boldsymbol{s}}^{\mathsf{T}} |\tilde{\boldsymbol{A}}_j \Delta \boldsymbol{x}|^2} - y_j\right)^2 \\ =& \sum_{i=1, i\neq j}^{M} f_i(\boldsymbol{x}) + \frac{1}{2K}\left((\alpha - \alpha^\star)\sqrt{\tilde{\boldsymbol{s}}^{\mathsf{T}} |\tilde{\boldsymbol{A}}_j \Delta \boldsymbol{x}|^2} - y_j\right)^2 \\ \leq& \sum_{i=1, i\neq j}^{M} f_i(\boldsymbol{x}) + \frac{1}{2K}\left((\alpha - \alpha^\star)\sqrt{\tilde{\boldsymbol{s}}^{\mathsf{T}} |\tilde{\boldsymbol{A}}_j \Delta \boldsymbol{x}|^2} + y_j\right)^2 \\ \leq& h(\alpha). \end{aligned} \tag{39}$$

As a result, we have

$$F(\boldsymbol{x}) = F(\boldsymbol{x} + \Delta \boldsymbol{x}) = g(1) \leq h(1) = Q(\boldsymbol{x}). \tag{40}$$

The above derivation can be easily extended to the case of passing through multiple nonsmooth points. With this, we conclude that for any $\boldsymbol{x} \in \mathbb{C}^n$, we have

$$F(\boldsymbol{x}) \leq Q(\boldsymbol{x}), \tag{41}$$

which completes the proof. □

Lemma 7. *The the Wirtinger flow iterates with* $\beta_t \equiv 0$ *converge to a stationary point using a fixed step size* $\gamma \leq 1/L$*, where* L *is the Lipschitz constant of* $\nabla F(\boldsymbol{x})$*.*

Proof. The proof is adapted from [4]. Recall that the non-accelerated Wirtinger flow iteration is given by

$$\boldsymbol{x}^{(t)} = \mathrm{prox}_{\gamma R}(\boldsymbol{x}^{(t-1)} - \gamma \nabla_{\boldsymbol{x}} F(\boldsymbol{x}^{(t-1)})). \tag{42}$$

According to Lemma 6, we have that

$$F(\boldsymbol{x}^{(t)}) \leq Q(\boldsymbol{x}^{(t)}) = F(\boldsymbol{x}^{(t-1)}) + \nabla F(\boldsymbol{x}^{(t-1)})^{\mathsf{H}}(\hat{\boldsymbol{x}}^{(t)} - \hat{\boldsymbol{x}}^{(t-1)}) + \frac{L}{2}\|\hat{\boldsymbol{x}}^{(t)} - \hat{\boldsymbol{x}}^{(t-1)}\|_2^2. \tag{43}$$

By the second prox theorem (Theorem 6.39) in [4], we have

$$(\hat{\boldsymbol{x}}^{(t-1)} - \gamma \nabla F(\boldsymbol{x}^{(t-1)}) - \hat{\boldsymbol{x}}^{(t)})^{\mathsf{H}}(\hat{\boldsymbol{x}}^{(t-1)} - \hat{\boldsymbol{x}}^{(t)}) \leq \gamma R(\boldsymbol{x}^{(t-1)}) - \gamma R(\boldsymbol{x}^{(t)}), \tag{44}$$

from which it follows that

$$\nabla F(\boldsymbol{x}^{(t-1)})^{\mathsf{H}}(\hat{\boldsymbol{x}}^{(t)} - \hat{\boldsymbol{x}}^{(t-1)}) \leq R(\boldsymbol{x}^{(t-1)}) - R(\boldsymbol{x}^{(t)}) - \frac{1}{\gamma}\|\hat{\boldsymbol{x}}^{(t-1)} - \hat{\boldsymbol{x}}^{(t)}\|_2^2. \tag{45}$$

Combining Eqs. (43) and (45), we arrive at

$$\begin{aligned} J(\boldsymbol{x}^{(t)}) \leq & J(\boldsymbol{x}^{(t-1)}) + \left(\frac{L}{2} - \frac{1}{\gamma}\right)\|\hat{\boldsymbol{x}}^{(t-1)} - \hat{\boldsymbol{x}}^{(t)}\|_2^2 \\ \leq & J(\boldsymbol{x}^{(t-1)}) - \frac{L}{2}\|\hat{\boldsymbol{x}}^{(t)} - \hat{\boldsymbol{x}}^{(t-1)}\|_2^2. \end{aligned} \tag{46}$$

Thus, the updating step for each iteration is upper-bounded:

$$\|\hat{\boldsymbol{x}}^{(t)} - \hat{\boldsymbol{x}}^{(t-1)}\|_2^2 \leq \frac{2}{L}(J(\boldsymbol{x}^{(t-1)}) - J(\boldsymbol{x}^{(t)})). \tag{47}$$

By summing up T iterations, we arrive at

$$\begin{aligned} \sum_{t=1}^{T} \|\hat{\boldsymbol{x}}^{(t)} - \hat{\boldsymbol{x}}^{(t-1)}\|_2^2 \leq & \frac{2}{L}\sum_{t=1}^{T}(J(\boldsymbol{x}^{(t-1)}) - J(\boldsymbol{x}^{(t)})) \\ \leq & \frac{2}{L}(J(\boldsymbol{x}^{(0)}) - J^{\star}), \end{aligned} \tag{48}$$

where $J^{\star} \geq 0$ denotes the global minimum value of the objective function. This implies that

$$\min_{t \in \{1,2,\ldots,T\}} \|\boldsymbol{x}^{(t)} - \boldsymbol{x}^{(t-1)}\|_2^2 \leq \frac{J(\boldsymbol{x}^{(0)}) - J^{\star}}{TL}, \tag{49}$$

and

$$\lim_{t \to \infty} \|\boldsymbol{x}^{(t)} - \boldsymbol{x}^{(t-1)}\|_2 = \mathbf{0}. \tag{50}$$

That is, the algorithm converges to a stationary point. □

Combining Lemma 5 and Lemma 7, we arrive at the main theorem below.

Theorem 1 (Convergence Theorem)**.** *The Wirtinger flow iterates with* $\beta_t \equiv 0$ *converge to a stationary point using a fixed step size* γ *that satisfies*

$$\gamma \leq 2K \Bigg/ \sum_{k=1}^{K} \rho\left(\boldsymbol{A}_k^{\mathsf{H}} \mathrm{diag}(\boldsymbol{s}) \boldsymbol{A}_k\right). \tag{51}$$

References

1. Horn, R.A.; Johnson, C.R. *Matrix analysis*; Cambridge university press, 2012.
2. Kreutz-Delgado, K. The complex gradient operator and the CR-Calculus. *arXiv preprint arXiv:0906.4835* **2009**.
3. Gao, Y.; Cao, L. Generalized optimization framework for pixel super-resolution imaging in digital holography. *Optics Express* **2021**, *29*, 28805–28823.
4. Beck, A. *First-order methods in optimization*; SIAM, 2017.
